# A facile synthesis, and antimicrobial and anticancer activities of some pyridines, thioamides, thiazole, urea, quinazoline, β-naphthyl carbamate, and pyrano[2,3-*d*]thiazole derivatives

**DOI:** 10.1186/s13065-018-0439-9

**Published:** 2018-06-20

**Authors:** Yasser H. Zaki, Marwa S. Al-Gendey, Abdou O. Abdelhamid

**Affiliations:** 1 Department of Chemistry, Faculty of Science, Beni-Suef University, Beni-Suef, 62514 Egypt; 2 Department of Chemistry, Faculty of Science and Humanity Studies at Al-Quwayiyah, Shaqra University, Al-Quwayiyah, 11971 Saudi Arabia; 3 Department of Chemistry, Faculty of Science (Girls Branch), Al-Azhar University, Cairo, Egypt; 4 Department of Chemistry, Faculty of Science, Cairo University, Giza, 12613 Egypt

**Keywords:** Antimicrobial, Anticancer, Pyridines, Thioamides, Thiazoles, Pyrano[2,3-*d*]thiazoles

## Abstract

**Background:**

Chalcones have a place with the flavonoid family and show a few very important pharmacological activities. They can used as initial compounds for synthesis of several heterocyclic compounds. The compounds with the backbone of chalcones have been reported to possess various biological activities.

**Results:**

Pyridine and thioamide derivatives were obtained from the reaction of 3-(furan-2-yl)-1-(*p*-tolyl)prop-2-en-1-one with the appropriate amount of malononitrile, benzoylacetonitrile, ethyl cyanoacetate and thiosemicarbazide in the presence of ammonium acetate. The reaction of 3,5-di(furan-2-yl)-4,5-dihydro-1*H*-pyrazole-1-carbothioamide with ethyl 2-chloro-3-oxobutanoate, 3-chloropentane-2,4-dione or ethyl chloroacetate produced thiazole derivatives. Pyrano[2,3-*d*]thiazole derivatives were obtained as well from thiazolone to arylidene malononitrile. The structures of the title compounds were clarified by elemental analyses, and FTIR, MS and NMR spectra. Some compounds were screened against various microorganisms (i.e., bacteria +ve, bacteria −ve and fungi). We observed that compounds (**3a**), (**4a**), (**4d**), (**5**), (**7**) and compound (**8**) exhibited high cytotoxicity against the MCF-7 cell line, with IC_50_ values of 23.6, 13.5, 15.1, 9.56, 14.2 and 23.5 μmol mL^−1^, respectively, while compound (**9**) was displayed the lowest values against MCF-7 cell lines.

**Conclusions:**

Efficient synthetic routes for some prepared pyridines, pyrazoline, thioamide, thiazoles and pyrano[2,3-d]thiazole were created. Moreover, selected newly-synthesized products were evaluated for their antitumor activity against two carcinoma cell lines: breast MCF-7 and colon HCT-116 human cancer cell lines.
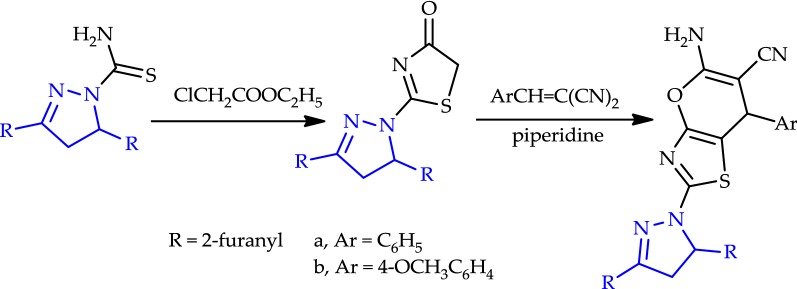

## Background

The chalcones (1,3-diaryl-2-propenones) and their derivatives are important intermediates in organic synthesis [1–3]. They serve as starting material for the synthesis of a variety of heterocyclic compounds of physiological importance. Due to the presence of enone functionality in chalcone, moiety confers antimicrobial [4–6], anti-inflammatory [7], antimalarial [8, 9], antileishmanial [10], antioxidant [11], antitubercular [12, 13], anticancer [14, 15] and other biological activities. In addition, thiazoles are involved in development of drugs for the treatment of allergies [16], hypertension [17], inflammation [18], schizophrenia [19], bacterial infections [20], HIV [21], sleep disorders [22] and, most recently, for of pain [23]. They function as fibrinogen receptor antagonists with antithrombotic activity [24], and as new inhibitors of bacterial DNA gyrase B [25]. In addition, pyrano[2,3-*d*]thiazoles are biologically interesting compounds with diabetes, obesity, hyperlipidemia, and atherosclerotic diseases [26]. They are also known to show antimicrobial, bactericidal, fungicidal and molluscicidal activities [27, 28]. In continuation of our previous work on the synthesis of new anticancer agents [29–34], we present here efficient syntheses of novel pyridines, pyrazolines, thiazoles and pyrano[2,3-*d*]thiazole derivatives which have not been previously reported. We investigated the anticarcinogenic effects against MCF-7, and the antibacterial activity of HCT-116 on human cancer cell lines against *Streptococcus pneumonia* and Bacillus *subtilis* as examples of Gram-positive bacteria and *Pseudomonas aeruginosa* and *Escherichia coli* as examples of Gram-negative bacteria.

## Results and discussion

### Chemistry

**Scheme 1:**
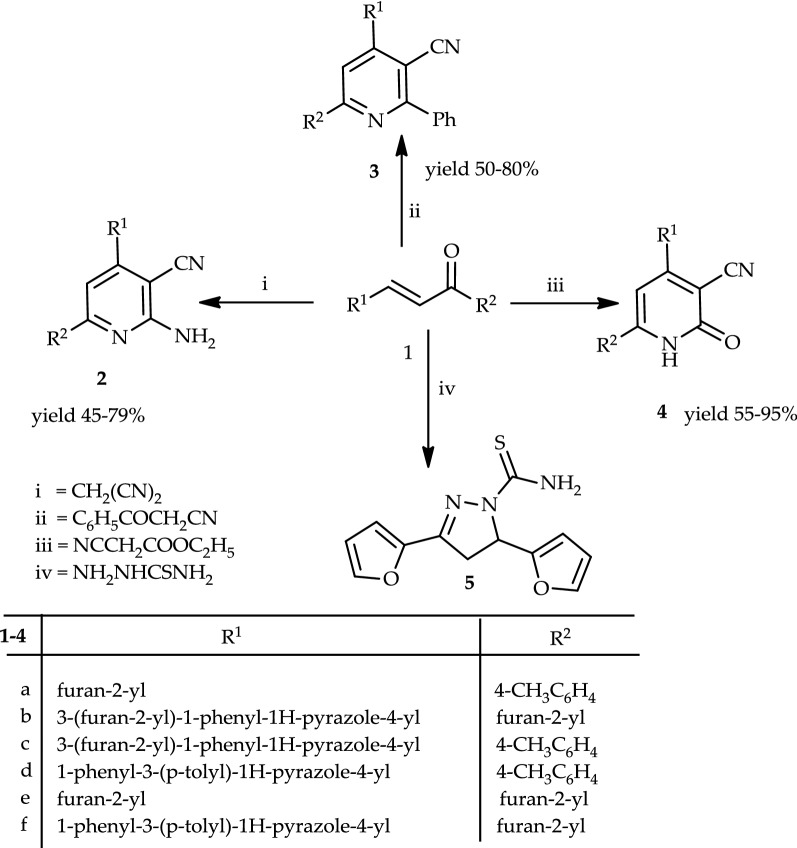
Synthesis of pyridine derivatives (**2**–**4**) and thioamide (**5**)

Reactions of 3-(furan-2-yl)-1-(*p*-tolyl)prop-2-en-1-one (**1a**) with an appropriate amount of malononitrile, benzoylacetonitrile, ethyl cyanoacetate, and thiosemicarbazide yielded 2-amino-4-(furan-2-yl)-6-(*p*-tolyl)nicotinonitrile (**2a**), 4-(furan-2-yl)-2-phenyl-6-(*p*-tolyl)nicotine-nitrile (**3a**), 4-(furan-2-yl)-2-oxo-6-(*p*-tolyl)-1,2-dihydropyridine-3-carbonitrile (**4a**), and 3,5-di(furan-2-yl)-4,5-dihydro-1*H*-pyrazole-1-carbothioamide (**5**), respectively (Scheme 1). Structures **2a**–**4a** and **5** were elucidated on the basis of elemental analyses and spectral data.

**Scheme 2:**
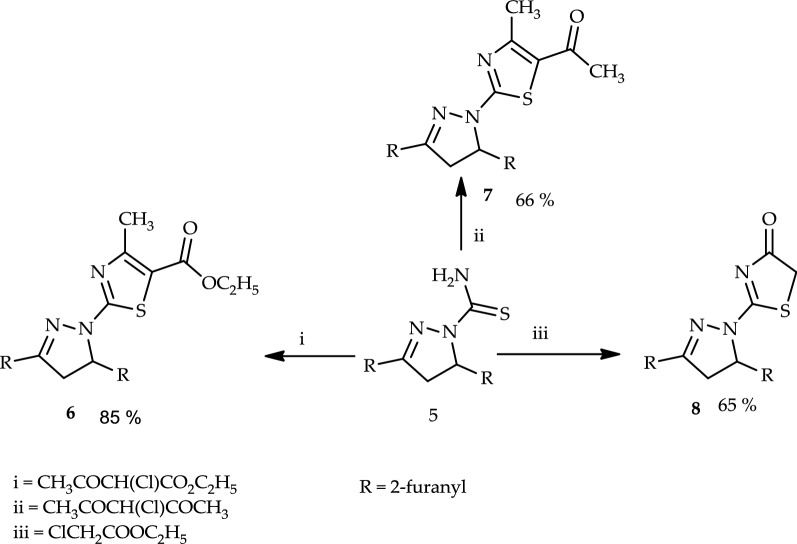
Synthesis of thiazole derivatives 6–8

Analogy, heating of the appropriate chalcone (**1b**–**f**) with malononitrile, benzoylacetonitrile, or ethyl cyanoacetate in glacial acetic acid in the presence of ammonium acetate created pyridine derivatives (**2**–**4**)**b**–**f** (*cf.* Scheme 1). Structures (**2**–**4**)**b**–**f** were elucidated by elemental analysis and spectral data (*cf*. “Experimental”). On the other hand, a reaction of 3,5-di(furan-2-yl)-4,5-dihydro-1*H*-pyrazole-1-carbothioamide (**5**), which was prepared from **1e** to thiosemicarbazide (each with ethyl 2-chloro-3-oxobutanoate, 3-chloropentane-2,4-dione, or ethyl 2-chloroacetate in ethanolic triethylamine) afforded ethyl 2-(3,5-di(furan-2-yl)-4,5-dihydro-1*H*-pyrazol-1-yl)-4-methylthiazole-5-carboxylate (**6**), 1-(2-(3,5-di(furan-2-yl)-4,5-dihydro-1*H*-pyrazol-1-yl)-4-methylthiazol-5-yl)ethan-1-one (**7**), and 2-(3,5-di(furan-2-yl)-4,5-dihydro-1*H*-pyrazol-1-yl)thiazol-4(5*H*)-one (**8**), respectively (Scheme 2). Structures (**6**–**8**) were confirmed with elemental analysis, spectral data, and chemical transformation.

**Scheme 3:**
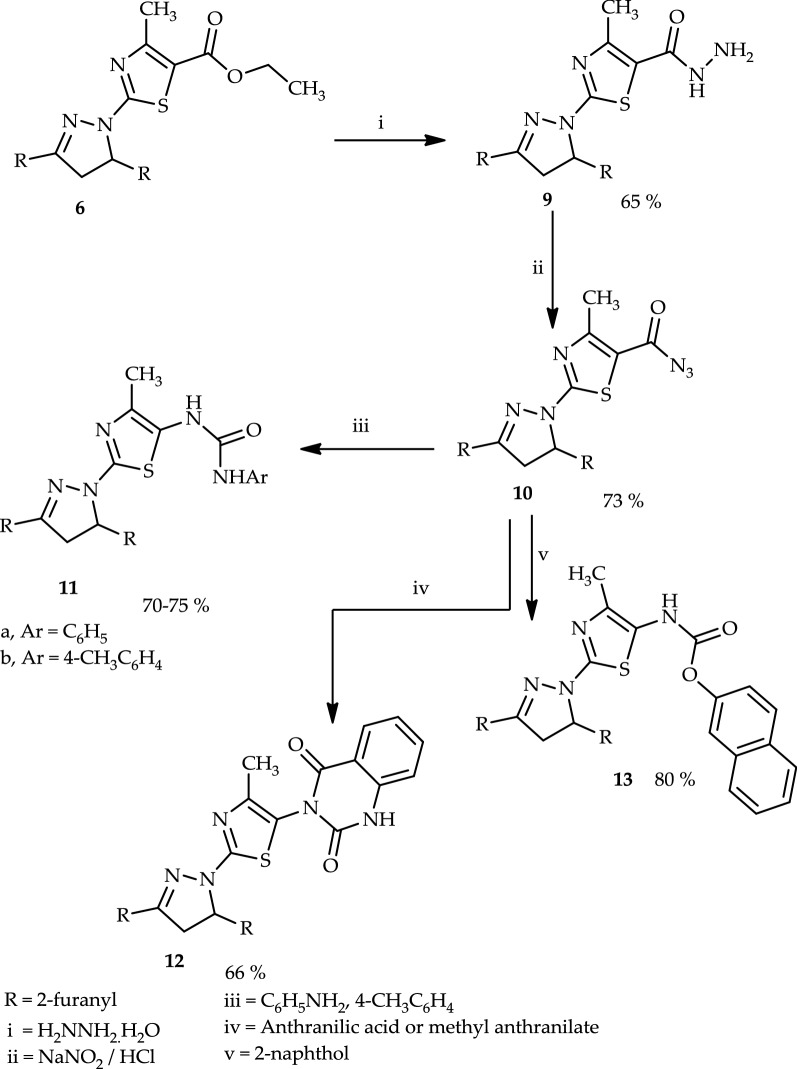
Synthesis of thiazole derivatives (**9**), (**10**), urea derivatives (**11a** and **11b**), quinazoline **12**, and β-naphthyl carbamate (**13**)

Compound (**6**) was further reacted with hydrazine hydrate afforded 2-(3,5-di(furan-2-yl)-4,5-dihydro-1*H*-pyrazol-1-yl)-4-methylthiazole-5-carbohydrazide (**9**) (Scheme 3). Structure **9** was elucidated by elemental analysis, spectra and chemical transformations. Thus, compound **9** reacted with nitrous acid yielded 2-(3,5-di(furan-2-yl)-4,5-dihydro-1*H*-pyrazol-1-yl)-4-methylthiazole-5-carbonyl azide (**10**). Structure **10** was confirmed by elemental analyses, spectral data and chemical transformation.

Treatment of compound **10** with each of the appropriate amounts of aniline, 4-toluidine, or anthranilic acid in boiling dioxane yielded 1-(2-(3,5-di(furan-2-yl)-4,5-dihydro-1*H*-pyrazol-1-yl)-4-methylthiazol-5-yl)-3-phenylurea (**11a**), 1-(2-(3,5-di(furan-2-yl)-4,5-dihydro-1*H*-pyrazol-1-yl)-4-methylthiazol-5-yl)-3-(*p*-tolyl)urea (**11b**), and 3-(2-(3,5-di(furan-2-yl)-4,5-dihydro-1*H*-pyrazol-1-yl)-4-methylthiazol-5-yl)quinazoline-2,4(1*H*, 3*H*)-dione (**12**), respectively. Additionally, compound **10** reacted with 2-naphthol in boiling benzene afforded naphthalen-2-yl(2-(3,5-di(furan-2-yl)-4,5-dihydro-1*H*-pyrazol-1-yl)-4-methylthiazol-5-yl)carbamate (**13**) (Scheme 3). The structure of compound **12** was confirmed by elemental analyses, spectral data, and an alternative synthetic route. Thus, compound **10** reacted with methyl anthranilate in dioxane afforded a product identical in all aspects (mp, mixed mp, and spectra) to compound **12**.

**Scheme 4:**
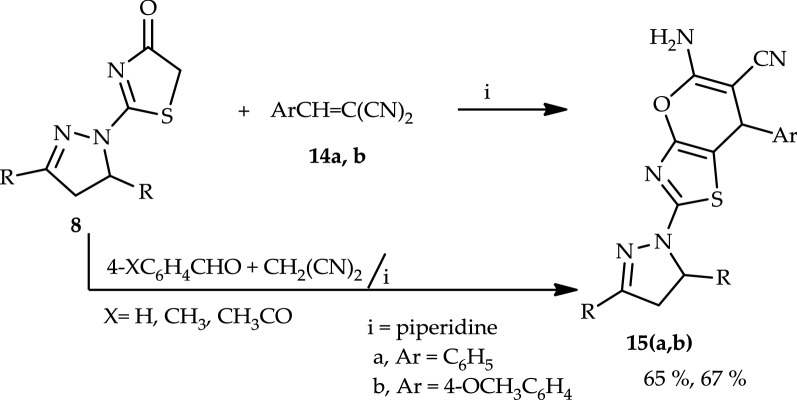
Synthesis of pyrano[2,3-*d*]thiazole derivatives (**15a** and **15b**)

Finally, treatment of compound **8** with benzylidenemalononitrile (**14a**) in refluxing ethanol containing a catalytic amount of piperidine afforded 5-amino-2-(3,5-di(furan-2-yl)-4,5-dihydro-1*H*-pyrazol-1-yl)-7-phenyl-7*H*-pyrano[2,3-*d*]thiazole-6-carbonitrile (**15a**) (Scheme 4). The structure of (**15a**) was elucidated by elemental analysis, spectral data, and a synthetic route. Furthermore, the infrared (IR) spectrum showed bands at 3388–3280 cm^−1^, which corresponded to the (NH_2_) group. Thus, a mixture of malononitrile, benzaldehyde, and 2-(3,5-di(furan-2-yl)-4,5-dihydro-1*H*-pyrazol-1-yl)thiazol-4(5*H*)-one (**8**) in ethanol containing a few drops of piperidine as a catalyst heated under reflux afforded a product identical in all aspects (mp, mixed mp, and spectra) with (**15a**). Similarly, compound **8** reacted with **14b** afforded 5-amino-2-(3,5-di(furan-2-yl)-4,5-dihydro-1*H*-pyrazol-1-yl)-7-(p-tolyl)-7*H*-pyrano[2,3-*d*]thiazole-6-carbonitrile (**15b**) (Scheme 4).

### Cytotoxicity evaluations

The in vitro growth inhibitory activity of the synthesized compounds **3a**, **4a**, **4d**–**4f**, **5**, **7**, **8**, **9**, **11a**, and **11b** was investigated against two carcinoma cell lines: breast MCF-7 and colon HCT-116 human cancer cell lines in comparison with the Imatinib anticancer standard drug (cisplatin) under the same conditions using the crystal violet viability assay. Data generated were used to plot a dose response curve where the concentration of test compounds required to kill 50% of the cell population (IC_50_) was determined and is summarized in Table 1. The IC_50_ values of the synthesized compounds **4a**, **4d**, **5**, **7,** and **8**, (IC_50_ = 9.65–23.6 μmol mL^−1^) were comparable to that of Imatinib. We observed that compounds **3a**, **4a**, **4d**, **5**, **7**, and **8** exhibited high cytotoxicity against the MCF-7 cell line, with IC_50_ values of 23.6, 13.5, 15.1, 9.56, 14.2 and 23.5 μmol/mL, respectively, while compound **9** was observed as having the lowest against the MCF-7 cell lines. Our results showed that compounds **4e**, **4f**, **11a** and **11b** had the lowest IC_50_ values against HCT-116 cancer cells.

**Table 1:** Cytotoxicity (IC_50_, μmol mL^−1^) of the synthesized compounds (**3a**–**11b**) against MCF-7 and HCT-116 human cancer cell lines

Compound no.	MCF-7	HCT-116	Compound no.	MCF-7	HCT-116
	IC_50_ (µmol mL^−1^)	IC_50_ (µmol mL^−1^)		IC_50_ (µmol mL^−1^)	IC_50_ (µmol mL^−1^)
**3a**	23.6	346	**7**	14.2	> 500
**4a**	13.5	291	**8**	23.5	> 500
**4d**	15.1	242	**9**	60.2	316
**4e**	222	193	**11a**	203	215
**4f**	238	124	**11b**	404	180
**5**	9.65	213			
Imatinib	24.5	–	Imatinib	24.5	–
Cisplatin		2.43	Cisplatin		2.43

### Antimicrobial activity

Nineteen of the newly synthesized target compounds were evaluated for their in vitro antibacterial activity against *Streptococcus pneumonia* and *Bacillus* subtilis (as examples of Gram-positive bacteria) and *Pseudomonas aeruginosa* and *Escherichia coli* (as examples of Gram-negative bacteria). They were also evaluated for their in vitro antifungal activity against a representative panel of fungal strains i.e., *Aspergillus fumigatus* and *Candida albicans* fungal strains. Ampicillin and Gentamicin are used as reference drugs for in vitro antibacterial activity while Amphotericin B is a reference drug for in vitro antifungal activity, respectively, at The Regional Center for Mycology and Biotechnology at Al-Azhar University (Nasr City, Cairo, Egypt). The results of testing for antimicrobial effects are summarized in Table 2.

**Table 2:** Mean zone of inhibition beyond well diameter (6 mm) produced on a range of clinically pathogenic microorganisms using a 5 mg mL^−1^ concentration of tested samples

Compound no.	*Aspergillus fumigatus* (fungus)	*Candida albicans* (fungus)	*Streptococcus pneumonia* (Gram +ve bact.)	*Bacillus subtilis* (Gram +ve bact.)	*Pseudomonas aeruginosa* (Gram −ve bact.)	*Escherichia coli* (Gram −ve bact.)
**2a**	15.4	14.8	10.9	12.9	17.3	11.6
**2b**	17.4	13.9	11.9	20.8	11.3	10.9
**2e**	14.8	11.9	15.1	16.3	11.1	11.4
**2f**	18.7	16.9	13.9	14.2	12.8	10.8
**3a**	12.7	15.2	14.1	12.8	0	10.1
**3b**	12.8	16.4	15.1	12.7	11.4	9.1
**3d**	14.8	11.9	13.2	13.5	13.8	12.6
**3e**	18.4	10.9	12.6	13.2	10.1	10.9
**3f**	15.7	15.9	16.7	19.2	0	13.6
**4a**	0.0	0.0	9.2	10.5	0	0
**4b**	17.7	18.4	15.7	15.3	13.2	9.6
**4c**	12.2	10.5	11.6	12.6	11.9	10.1
**4e**	15.4	10.4	10.9	12.9	11.3	11.6
**4f**	15.7	13.8	17.9	18.2	0	12.9
**6**	16.2	12.5	16.8	14.6	12.1	12.8
**11a**	19.1	16.9	13.6	14.7	12.1	10.4
**11b**	14.8	16.3	15.1	16.3	11.1	11.4
**12**	18.4	16.3	12.6	13.2	10.1	10.9
**13**	20.8	16.8	13.1	10.8	13.4	12.3
Amphotericin B	23.7	25.4	–	–	–	–
Ampicillin	–	–	23.8	32.4	–	–
Gentamicin	–	–	–	–	17.3	19.9

*Candida albicans* and *aspergillus fumigatus* were resistant to compound **4a**

*Pseudomonas aeruginosa* was resistant to compounds **3a**, **3f**, **4a**, and **4f**

*Aspergillus fumigatus* was susceptible to compounds to **2b**, **2f**, **3e**, **4b**, **11a**, **12** and **13** while being moderate to **2a**, **2e**, **3a**–**3d**, **3f**, **4c**, **4e**–**4f**, **6**, and **11b** when compared to the Amphotericin B standard

*Candida albicans* was moderate to all compounds except **4a** when compared to the Amphotericin B standard

*Streptococcus pneumoniae* was moderate to all compounds when compared to the Ampicillin standard

*Bacillus subtilis* was moderate to all compounds when compared to the Ampicillin standard

*Pseudomonas aeruginosa* was moderate to all compounds except compounds **3a**, **3f**, **4a**, and **4f**, which were resistant to when compared to their standard Gentamicin

*Escherichia coli* was moderate to all compounds except **4a**, which was resistant when compared to the Gentamicin standard

### Experimental section

#### General information

All melting points were measured with a Gallenkamp melting point apparatus (Weiss–Gallenkamp, London, UK). The infrared spectra were recorded using potassium bromide disks on pye Uni-cam SP 3300 and Shimadzu FT-IR 8101 PC infrared spectrophotometers (Pye Unicam Ltd. Cambridge, England, and Shimadzu, Tokyo, Japan, respectively). The NMR spectra were recorded on a Varian Mercury VX-300 NMR spectrometer (Varian, Palo Alto, CA, USA). ^1^H spectra were run at 300 MHz and ^13^C spectra were run at 75.46 MHz in deuterated chloroform (CDCl_3_) or dimethyl sulphoxide (DMSO-d6). Chemical shifts were related to that of the solvent. Mass spectra were recorded on a Shimadzu GCMS-QP 1000 EX mass spectrometer (Shimadzu) at 70 eV. Elemental analyses were carried out at the Microanalytical Center of Cairo University. The antimicrobial and ant-cancer screening was performed at the Regional Center for Mycology and Biotechnology, Al-Azhar University, Cairo, Egypt.

#### General methods for the synthesis of pyridines (**2**–**4**)**a**–**f**

*Method A* A mixture of the appropriate chalcones (**1a**–**f**) (10 mmol), and the appropriate amount of malononitrile, benzoylacetonitrile, or ethyl cyanoacetate (10 mmol) in glacial acetic acid containing ammonium acetate (0.77 g, 10 mmol) was refluxed for 3–4 h, and the acetic acid was evaporated under reduced pressure, left to cool, then poured.

gradually with stirring onto crushed ice. The solid formed was filtered off, dried, and recrystallized from an appropriate solvent to obtain the corresponding pyridines (**2**–**4**)**a**–**f**, respectively.

*Method B* A mixture of the appropriate aldehydes (10 mmol), arylketone (10 mmol), and the appropriate amount of malononitrile, benzoylacetonitrile, or ethyl cyanoacetate (10 mmol) in *n*-butanol (20 mL) containing ammonium acetate (6.00 g, 77 mmol) was refluxed for 3–4 h, then the solvent evaporated under reduced pressure, left to cool, then poured gradually with stirring onto crushed ice. The solid formed was filtered off, dried, and recrystallized from an appropriate solvent to obtain products that were identical in all respects (mp, mixed mp, and IR spectra) with the corresponding pyridines (**2**–**4**)**a**–**f**, respectively. The products (**2**–**4**)**a**–**f** together with their physical constants are listed below.

##### 2-Amino-4-(furan-2-yl)-6-(*p*-tolyl)nicotinonitrile (**2a**)

Pale yellow solid from glacial acetic acid, yield (1.79 g, 65%), mp: 259–260 °C; IR (KBr, cm^−1^): 3304, 3260 (NH_2_), 3145 (= C–H), 2914 (–C–H), 2208 (–CN), 1647 (–C=N); ^1^H NMR (CDCl_3_): $\delta$ 2.46 (s, 3H, 4-CH_3_C_6_H_4_), 6.63 (t, 1H, *J* = 4 Hz, furan H-4), 7.17 (s, 1H, pyridine H-5), 7.22–7.25 (m, 3H, ArH’s and furan H-3), 7.40 (s, br., 2H, NH_2_), 7.58–7.59 (d, 1H, *J* = 4 Hz, furan H-5), 7.65–7.68 (m, 2H, ArH’s); ^13^C-NMR (DMSO-*d*6) δ 21.4 (CH_3_), 87.7, 110.2, 110.5, 115.4, 116.9, 127.4, 129.4, 133.1, 137.2, 143, 146.5, 150.7, 156.9, 1159.1; MS (*m/z*): 275 (M^+^, 1), 274 (9), 240 (43), 212 (19), 169 (34), 141 (35), 169 (34), 141 (35), 108 (28), 107 (21), 91 (9), 79 (31), 44 (100); *Anal.* Calcd. for C_17_H_13_N_3_O (275.30): C, 74.17; H, 4.76; N, 15.26; found: C, 74.21; H, 4.64; N, 15.15.

##### 2-Amino-6-(furan-2-yl)-4-(3-(furan-2-yl)-1-phenyl-*1H*-pyrazol-4-yl)nicotinonitrile (**2b**)

Yellow solid from glacial acetic acid, yield (2.8 g, 72%), mp: 183–184 °C; IR (KBr, cm^−1^): 3327, 3265 (NH_2_), 3055 (= C–H), 2208 (–CN), 1647 (–C=N); ^1^H NMR (CDCl_3_): $\delta$: 6.71 (t, 1H, furan H-4′), 7.14–7.16 (d, 1H, furan H-3), 7.48–7.96 (m, 12H, ArH’s, NH_2_, furan H’s and pyridine H-5), 9.15 (s, 1H, pyrazole H-5); ^13^C-NMR (DMSO-*d*6) δ: 90.1, 112.0, 112.1, 114.1, 114.3, 115.2, 116.9, 117.6, 120.3, 127.5, 128.3, 129,5, 137.4, 140.8, 141.3, 141.7, 143.5, 144.7, 148.7, 150.2, 159.4; MS (*m/z*): 393 (M+, 1), 376 (7), 358 (10), 334 (1), 316 (24), 298 (40), 270 (17), 255 (24), 241 (14), 227 (16), 212 (13), 201 (15), 187 (16), 171 (14), 159 (17), 135 (20), 109 (20), 91 (22), 69 (23), 43 (100); Anal. Calcd. for C_23_H_15_N_5_O_2_ (393.40): C, 70.22; H, 3.84; N, 17.80; found: C, 70.36; H, 3.84; N, 17.94.

##### 2-Amino-4-(3-(furan-2-yl)-1-phenyl-*1H*-pyrazol-4-yl)-6-(p-tolyl)nicotinonitrile (**2c**)

Yellow solid from glacial acetic acid, yield (3.09 g, 74%), mp: 200–203 °C; IR (KBr, cm^−1^): 3307, 3275 (–NH_2_), 2924 (–C–H), 2192 (–CN); ^1^H NMR (CDCl_3_): $\delta$: 2.44 (s, 3H, 4-CH_3_C_6_H_4_), 5.22 (s, br., 2H, NH_2_), 6.33–7.55 (m, 13H, ArH’s + furan H’s + pyridine H-5), 9.45 (s, 1H, pyrazole H-5); ^13^C-NMR (DMSO-*d*6) δ: 21.4 (CH_3_), 91.6, 112.1, 113.5, 115.5, 116.9, 117.6, 120.3, 127.6, 128.1, 129.3, 129.6, 131.3, 137.1, 138.0, 140.9, 141.3, 143.4, 150.2, 158.3, 158.6; MS (*m/z*): 419 (M+2, 4), 418 (M+1, 23), 417 (M+, 100), 222 (60), 195 (70), 180 (48), 166 (6), 152 (8), 94 (6), 77 (2), 43 (15); *Anal.* Calcd. for C_26_H_19_N_5_O (417.46): C, 74.80; H, 4.59; N, 16.78; found: C, 74.92; H, 4.70; N, 16.67.

##### 2-Amino-4-(1-phenyl-3-(p-tolyl)-*1H*-pyrazol-4-yl)-6-(p-tolyl)nicotinonitrile (**2d**)

Yellow solid from benzene, yield (3.48 g, 79%), mp: 225–227 °C; IR (KBr, cm^−1^): 3348, 3240 (NH_2_), 3039 (=C–H), 2920 (–C–H), 2214 (–CN); ^1^H NMR (CDCl_3_): $\delta$: 2.39 (s, 3H, 4-CH_3_C_6_H_4_), 2.43 (s, 3H, 4-CH_3_C_6_H_4_), 5.22 (s, br., 2H, NH_2_), 7.24–7.82 (m, 14H, ArH’s + pyridine H-5), 8.40 (s, 1H, pyrazole H-5); ^13^C-NMR (DMSO-*d*6) δ: 21.4 (2CH_3_), 91.7, 113.2, 115.2, 116.9, 120.3, 127.5, 127.7, 129.0, 129.3, 129.5, 129.6, 130.7, 133.1, 134.7, 136.2, 137.2, 137.4, 138.1, 141.3, 149.8, 158.3, 158.7; MS (*m/z*): 443 (M+2, 0.51), 442 (M+1, 0.6), 441 (M+, 0.48), 426 (31), 425 (100), 411 (6), 400 (6), 334 (10), 308 (3), 334 (10), 308 (3), 259 (8), 104 (16), 91 (30), 77 (94), 64 (42); *Anal.* Calcd. for C_29_H_23_N_5_ (441.53): C, 78.89; H, 5.25; N, 15.86; found: C, 78.95; H, 5.18; N, 15.63.

##### 2-Amino-4,6-di(furan-2-yl)nicotinonitrile (**2e**)

Yellow solid from glacial acetic acid, yield (1.13 g, 45%), mp: 213–215 °C; IR (KBr, cm^−1^): 3374, 3298 (NH_2_), 3008 (=C–H); ^1^H NMR (CDCl_3_): $\delta$: 6.24-6.27 (t, 1H, furan H-4), 6.53–6.54 (t, 1H, furan H-4′), 6.89–7.00 (d, 1H, furan H-2), 7.11–7.12 (d, 1H, furan H-5′), 7.22 (s, 1H, pyridine H-4), 7.24–7.25 (d, 1H, furan H-3), 7.40 (s, br., 2H, NH_2_), 8.10 (d, 1H, furan H-5); ^13^C-NMR (DMSO-*d*6) δ: 94.1, 96.8, 105.8, 107.45, 114.6, 115.4, 115.7, 142.3, 143.4, 147.5, 151.3, 151.9, 152.9, 165.3. MS (*m/z*): 251 (M+, 3), 238 (52), 181 (23), 178 (86), 152 (19), 149 (23), 122 (18), 117 (15), 104 (27), 83 (44), 79 (16), 77 (18), 43 (100); *Anal.* Calcd. for C_14_H_9_N_3_O_2_ (251.24): C, 66.93; H, 3.61; N, 16.73; found: C, 66.80; H, 3.72; N, 16.64.

##### 2-Amino-6-(furan-2-yl)-4-(1-phenyl-3-(*p*-tolyl)-*1H*-pyrazol-4-yl)nicotinonitrile (**2f**)

Yellow solid from glacial acetic acid, yield (2.75 g, 66%), mp: 208–211 °C; IR (KBr, cm^−1^): 3384, 3294 (NH_2_), 2920 (–C–H), 2200 (–CN), 1600 (–C=N); ^1^H NMR (CDCl_3_): $\delta$: 2.30 (s, 3H, 4-CH_3_C_6_H_4_), 6.27-6.28 (t, 1H, furan H-4), 6.89–6.99 (d, 1H, furan H-3), 7.02 (s, 1H, pyridine H-5), 7.11-7.13 (d, 1H, furan H-2), 7.23-7.94 (m, 11H, ArH’s + NH_2_ + furan- H’s), 9.41 (s, 1H, pyrazole H-4); ^13^C-NMR (DMSO-*d*6) δ: 21.4 (CH_3_), 90.8, 112.1, 114.3, 1146, 115.2, 120.3, 127.5, 129.0, 129.2, 129.5, 134.7, 136.4, 137.4, 141.2, 141.5, 144.5, 148.7, 149.8, 159.6; MS (*m/z*): 418 (M+1, 23), 417 (M+, 100), 223 (12), 222 (60), 196 (98), 195 (70), 194 (15), 131 (38), 180 (48), 152 (8), 43 (15); *Anal.* Calcd. for C_26_H_19_N_5_O (417.46): C, 74.80; H, 4.59; N, 16.78; found: C, 74.71; H, 4.65; N, 16.94.

##### 4-(Furan-2-yl)-2-phenyl-6-(*p*-tolyl)nicotinonitrile (**3a**)

Yellow solid from glacial acetic acid, yield (2.15 g, 64%), mp: 155–156 °C; IR (KBr, cm^−1^): 3024 (=C–H), 3062, 2916 (–C–H), 2214 (–CN); ^1^H NMR (CDCl_3_): $\delta$: 2.44 (s, 3H, 4-CH_3_C_6_H_4_), 6.64–6.66 (d, 1H, furan H-4), 7.21 (s, 1H, pyridine H-5), 7.27–7.83 (m, 9H, ArH’s and furan H-3, H-5), 8.44–8.46 (d, 2H, ArH’s); ^13^C-NMR (DMSO-*d*6) δ: 21.4 (CH_3_), 106.8, 110.3,113.5 120.3, 125.6, 126.4, 127.5, 132.6, 138.3, 139.6, 142.5, 157.9, 171.7, 177.3, 183.9; MS (*m/z*): 337 (M+1, 2), 336 (M+, 12), 245 (6), 230 (10), 202 (9), 180 (6), 158 (5), 132 (18), 65 (14); *Anal.* Calcd. for C_23_H_16_N_2_O (336.39): C, 82.12; H, 4.79; N, 8.33; found: C, 82.00; H, 4.67; N, 8.45.

##### 6-(Furan-2-yl)-4-(3-(furan-2-yl)-1-phenyl-*1H*-pyrazol-4-yl)-2-phenylnicotinonitrile (**3b**)

White solid from glacial acetic acid, yield (3.22 g, 71%), mp: 199–200 °C; IR (KBr, cm^−1^): 3052 (=C–H), 2210 (–CN); ^1^H NMR (CDCl_3_): $\delta$: 6.60–6.61 (t, 1H, furan H-3), 6.77–6.81 (m, 3H, furan H’s), 7.12 (s, 1H, pyridine H-5), 7.42–8.00 (m, 12H, ArH’s + furan–H’s), 9.63 (s, 1H, pyrazole H-5); ^13^C-NMR (DMSO-*d*6) δ: 104.3, 105.4, 105.9, 109.5, 110.5, 112.7, 126.6, 118.7, 122.2, 123.9, 124.5, 129.7, 130.8, 137.6, 142.7, 140.6, 143.5, 149.8, 152.1, 153.6, 154.7, 163.7; MS (*m/z*): 455 (M+1, 2), 454 (M+, 8), 382 (16), 323 (24), 262 (93), 220 (55), 203 (19), 194 (41), 177 (21), 147 (31), 133 (52), 121 (37), 107 (56), 91 (16), 73 (66), 69 (100), 41 (42), 30 (49); *Anal.* Calcd. for C_29_H_18_N_4_O_2_ (454.48): C, 76.64; H, 3.99; N, 12.33; found: C, 76.52; H, 4.16; N, 12.28.

##### 4-(3-(Furan-2-yl)-1-phenyl-*1H*-pyrazol-4-yl)-2-phenyl-6-(*p*-tolyl)nicotinonitrile (**3c**)

White solid from glacial acetic acid, yield (3.59 g, 75%), mp: 202–203 °C; IR (KBr, cm^−1^): 3040 (=C–H), 2919 (–C–H), 2213 (–CN); ^1^H NMR (CDCl_3_): $\delta$: 2.43 (s, 3H, 4-CH_3_C_6_H_4_), 6.52 (t, 1H, furan H), 6.76 (t, 1H, furan H), 7.16 (s, 1H, pyridine H-5), 7.27–8.07 (m, 15H, ArH’s), 8.39 (s, 1H, pyyrazole H-5); ^13^C-NMR (DMSO-*d*6) δ: 21.4 (CH_3_), 100.2, 104.4, 112.4, 115.3, 118.6, 121.1, 122.2, 123.8, 124.3, 126.4, 129.7, 130.7, 136.6, 137.9, 139.7, 142.1, 142.8, 149.7, 154.9, 160.5, 163.3; MS (*m/z*): 480 (M+1, 4), 479 (M+, 24), 478 (87), 449 (27), 321 (24), 304 (18), 277 (25), 249 (41), 322 (23), 219 (14), 205 (25), 179 (13), 166 (28), 152 (56), 29 (100); *Anal.* Calcd. for C_32_H_22_N_4_O (478.54): C, 80.32; H, 4.63; N, 11.71; found: C, 80.15; H, 4.50; N, 11.84.

##### 2-Phenyl-4-(1-phenyl-3-(p-tolyl)-*1H*-pyrazol-4-yl)-6-(p-tolyl)nicotinonitrile (**3d**)

White solid from glacial acetic acid, yield (4.02 g, 80%), mp: 216–217 °C; IR (KBr, cm^−1^): 3033 (=C–H), 2915 (–C–H), 2211 (–CN); ^1^H NMR (CDCl_3_): $\delta$: 2.41 (s, 3H, 4-CH_3_C_6_H_4_), 2.43 (s, 3H, 4-CH_3_C_6_H_4_), 7.25 (s, 1H, pyridine H-5), 7.22–8.03 (m, 18H, ArH’s), 8.53 (s, 1H, pyrazole H-5); ^13^C-NMR (DMSO-*d*6) δ: 21.0 (CH_3_), 21.4 (CH_3_), 109.3, 115.3, 116.8, 120.4, 124.4, 126.6, 127.2, 127.5, 127.8, 129.4, 131.08, 133.9, 133.9, 136.3, 137.7, 139.1, 139.3, 142.5, 148.9, 169.1, 175.2, 188.5; MS (*m/z*): 504 (M+2, 0.5), 503 (M+1, 2.7), 502 (M+, 7.7), 259 (37), 251 (9), 234 (4), 214 (2), 79 (100), 77 (25), 65 (9), 63 (51), 60 (24), 57 (6); *Anal.* Calcd. for C_35_H_26_N_4_ (502.61): C, 83.64; H, 5.21; N, 11.15; found: C, 83.52; H, 5.32; N, 11.06.

##### 4,6-Di(furan-2-yl)-2-phenylnicotinonitrile (**3e**)

White solid from glacial acetic acid, yield (1.74 g, 56%), mp: 213–214 °C; IR (KBr, cm^−1^): 3151; 3055 (=C–H), 2215 (CN); ^1^H NMR (CDCl_3_): $\delta$: 6.74 (t, 1H, furan H-3), 6.75 (t, 1H, furan H-3′), 7.30 (s, 1H, pyridine H-5), 7.40–8.00 (m, 7H, ArH’s + furyl-H’s), 8.10–8.12 (d, 2H, ArH’s); ^13^C-NMR (DMSO-*d*6) δ: 101.6, 108.6, 109.5, 110.8, 112.0,121.4, 126.5, 126.9, 134.8, 141.3, 142.6, 143.5, 156.7, 157.8, 171.6, 177.6, 197.7. MS (*m/z*): 314 (M+2, 0.2), 313 (M 1, 1.7), 312 (M+, 100), 294 (55), 299 (88), 239 (42), 223 (19), 210 (17), 197 (18), 179 (13), 167 (18), 110 (21), 81 (20), 55 (45), 41 (25); Anal. Calcd. for C_20_H_12_N_2_O_2_ (312.32): C, 76.91; H, 3.87; N, 8.97; found: C, 76.83; H, 3.79; N, 9.12.

##### 6-(Furan-2-yl)-2-phenyl-4-(1-phenyl-3-(*p*-tolyl)-*1H*-pyrazol-4-yl)nicotinonitrile (**3f**)

White solid from glacial acetic acid, yield (2.39 g, 50%), mp: 186–187 °C; IR (KBr, cm^−1^): 3056 (=C–H), 2917 (–C–H), 2215 (–CN); ^1^H NMR (CDCl_3_): $\delta$: 2.48 (s, 3H, 4-CH_3_C_6_H_4_), 6.18–6.20 (t, 1H, furan H-4), 6.88-6.89 (d, 1H, furan H-5), 7.9 (s, 1H, pyridine H-5), 7.31–7.85 (m, 13H, ArH’s + furan-H’s), 8.44–8.45 (d, 2H, ArH’s), 9.24 (s, 1H, pyrazole H-5); ^13^C-NMR (DMSO-*d*6) δ: 101.3, 108.2, 108.8, 109.6, 110.7, 111.8, 121.4, 126.6, 126.8, 134.7, 141.2, 142.5, 143.3, 131.8, 156.3, 158.2, 137.7, 171.5, 177.4, 180.1; MS (*m/z*): 478 (M+, 5), 256 (10), 225 (12), 161 (12), 135 (19), 134 (12), 123 (14), 122 (100), 121 (73), 119 (11), 107 (13), 91 (19), 77 (10), 55 (17), 28 (17); Anal. Calcd. for C_32_H_22_N_4_O (478.54): C, 80.32; H, 4.63; N, 11.71; found: C, 80.43; H, 4.54; N, 11.88.

##### 4-(Furan-2-yl)-2-oxo-6-(*p*-tolyl)-1,2-dihydropyridine-3-carbonitrile (4a)

White solid from dioxane, yield (2.62 g, 95%), mp: 305–306 °C; IR (KBr, cm^−1^): 3350 (N–H), 3016 (=C–H), 2912 (–C–H), 2218 (–CN), 1654 (–C=O); ^1^H NMR (CDCl_3_): $\delta$: 2.38 (s, 3H, 4-CH_3_C_6_H_4_), 6.83 (t, 1H, Furyl H-5), 7.19 (s, 1H, pyridine H-5), 7.02–7.45 (m, 5H, ArH’s + furyl-H’s), 8.03–8.05 (d, 1H, furan H-5), 12.54 (s, 1H, N–H); ^13^C-NMR (DMSO-*d*6) δ: 21.2 (CH_3_), 90.4, 120.2, 112.4, 115.7, 117.9, 126.3, 128.3, 134.3, 140.4, 142.6, 143.2, 146.4, 154.3, 158.4; MS (*m/z*): 278 (M+2, 1), 277 (M+1, 15), 276 (M+, 100), 241 (9), 97 (55), 77 (20), 67 (24), 41 (8); *Anal.* Calcd. for C_17_H_12_N_2_O_2_ (276.29): C, 73.90; H, 4.38; N, 10.14; found: C, 74.10; H, 4.52; N, 10.31.

##### 6-(Furan-2-yl)-4-(3-(furan-2-yl)-1-phenyl-*1H*-pyrazol-4-yl)-2-oxo-1,2-dihydropyridine-3-carbonitrile (**4b**)

Yellow solid from glacial acetic acid, yield (3.47 g, 88%), mp: 319–320 °C; IR (KBr, cm^−1^): 3269 (N–H), 3123 (=C–H), 2919 (–C–H), 2216 (–CN), 1683 (–C=O); ^1^H NMR (CDCl_3_): $\delta$: 6.53–6.59 (t, 1H, furan H-4), 6.75–6.77 (m, 2H, furan H-4′, H-3), 7.38–7.79 (m, 8H, ArH’s + furan-H’s), 8.22 (s, 1H, pyridine H-5), 8.38 (s, 1H, pyrazole H−=5), 11.35 (s, 1H, NH); ^13^C-NMR (DMSO-*d*6) δ: 86.4, 89.8, 105.0, 109.6, 111.1, 113.6, 118.9, 119.6, 123.2, 124.1, 126.2, 129.3, 134.5, 137.9, 139.2, 140.1, 144.6, 144.9, 145.2, 149.2, 156.9; MS (*m/z*): 395 (M+1, 1), 394 (M+, 6), 393 (49), 379 (29), 364 (8), 351 (8), 133 (9), 119 (11), 107 (33), 91 (100), 77 (8), 65 (19); *Anal.* Calcd. for C_23_H_14_N_4_O_3_ (394.38): C, 70.05; H, 3.58; N, 14.21; found: C, 70.23; H, 3.50; N, 14.00.

##### 4-(3-(Furan-2-yl)-1-phenyl-*1H*-pyrazol-4-yl)-2-oxo-6-(*p*-tolyl)-1,2-dihydropyridine-3-carbonitrile (**4c**)

Pale yellow solid from dioxane, yield (3.89 g, 93%), mp: 339–340 °C; IR (KBr, cm^−1^): 3425 (N–H), 3105 (=C–H), 2905 (–C–H), 2214 (–CN), 1644 (–C=O); ^1^H NMR (CDCl_3_): $\delta$: 2.45 (s, 3H, 4-CH_3_C_6_H_4_), 6.73 (t, 1H, furan H-4), 6.67–6.68 (d, 1H, furan H-3), 7.72–7.82 (m, 10H, ArH’s + furan H-5), 7.94 (s, 1H, pyridine H-5), 8.42 (s, 1H, pyrazole H-5), 11.61 (s, 1H, NH);); ^13^C-NMR (DMSO-*d*6) δ: 21.2 (CH_3_), 87.1, 88.1, 105.1, 109.4, 118.9, 120.3, 123.3, 124.4, 124.8, 127.3, 129.2, 136.8, 137.8, 137.8, 139.4, 140.2, 145.5, 149.2, 157.9, 163.5; MS (*m/z*): 418 (M+, 6), 280 (10), 256 (50), 245 (32), 163 (19), 120 (16), 91 (16), 61 (24), 43 (100), 31 (47), 15 (17); *Anal.* Calcd. for C_26_H_18_N_4_O_2_ (418.45): C, 74.63; H, 4.34; N, 13.39; found: C, 74.50; H, 4.51; N, 13.61.

##### 2-Oxo-4-(1-phenyl-3-(p-tolyl)-*1H*-pyrazol-4-yl)-6-(*p*-tolyl)-1,2-dihydropyridine-3-carbonitrile (**4d**)

White solid from glacial acetic acid, yield (3.76 g, 85%), mp: 325–326 °C; IR (KBr, cm^−1^): 3441 (N–H), 3131 (=C–H aromatic), 3016 (=C–H), 2914 (–C–H), 2215 (–CN), 1640 (–C=O); ^1^H NMR (CDCl_3_): $\delta$: 2.40 (s, 3H, 4-CH_3_C_6_H_4_), 2.45 (s, 3H, 4-CH_3_C_6_H_4_), 7.27–7.46 (m, 10 H, ArH’s), 7.64–7.97 (m, 4H, ArH’s and pyridine H-5), 9.23 (s, 1H, pyrazole H-5), 11.61 (s, 1H, NH); ^13^C-NMR (DMSO-*d*6) δ: 21 (CH_3_), 21.4 (CH_3_), 86.20, 87.60, 119.4, 123.6, 127.5, 127.7, 128.4, 129.2,129.7, 136.6, 139.5, 140.6, 144.5, 150.3,150.8, 157.9, 164.1; MS (*m/z*): 443 (M+1, 5), 442 (M+, 28), 441 (28), 424 (14), 415 (100), 397 (7), 295 (5), 268 (4), 199 (7), 191 (5), 140 (4), 118 (16), 104 (8), 91 (24), 77 (55), 63 (25), 51 (12); *Anal.* Calcd. for C_29_H_22_N_4_O (442.51): C, 78.71; H, 5.01; N, 12.66; found: C, 78.66; H, 5.18; N, 12.77.

##### 4,6-Di(furan-2-yl)-2-oxo-1,2-dihydropyridine-3-carbonitrile (**4e**)

White solid from dioxane, yield (1.38 g, 55%), mp: 342–343 °C; IR (KBr, cm^−1^): 3445 (N–H), 3115 (=C–H), 2216 (–CN), 1640 –C=O); ^1^H NMR (CDCl_3_): $\delta$: 6.66–6.68 (t, 1H, furan H-4), 6.72 (d, 1H, furan H-3), 6.82–6.84 (t, 1H, furan H-3′), 7.16-7.25 (m, 4H, furan H’s + pyridine H-5, furan H’s), 11.63 (s, 1H, N–H); ^13^C-NMR (DMSO-*d*6) δ: 14.0, 58.6, 98.8, 102.5, 103.6. 106.8, 115.6, 120.3, 141.9, 142.5, 143.4, 143.9, 151.3, 156.8, 159.7, 196.8. MS (*m/z*): 252 (M+, 4), 249 (16), 245 (16), 218 (13), 203 (11), 184 (17), 173 (18), 171 (91), 156 (29), 155 (14), 144 (18), 129 (35), 115 (26), 91 (14), 28 (100); *Anal.* Calcd. for C_14_H_8_N_2_O_3_ (252.22): C, 66.67; H, 3.20; N, 11.11; found: C, 66.78; H, 3.00; N, 11.25.

##### 6-(Furan-2-yl)-2-oxo-4-(1-phenyl-3-(p-tolyl)-*1H*-pyrazol-4-yl)-1,2-dihydropyridine-3-carbonitrile (**4f**)

Pale yellow solid from dioxane, yield (3.76 g, 90%), mp: 311–313 °C; IR (KBr, cm^−1^): 3421 (N–H), 3118 (=C–H), 2911 (–C–H), 2213 (–CN), 1648 (–C=O); ^1^H NMR (CDCl_3_): $\delta$: 2.50 (s, 3H, 4-CH_3_C_6_H_4_), 6.63-6.65 (t, 1H, furan H-4), 6.72–6.74 (d, 1H, furan H-3), 7.22–7.55 (m, 6H, ArH’s and furan H-5), 7.79–7.81 (d, 2H, ArH’s), 8.03–8.05 (d, 2H, ArH,s), 8.22 (s, 1H, pyridine H-5), 8.35 (s, 1H, pyrazole H-5), 11.62 (s, 1H, NH);); ^13^C-NMR (DMSO-*d*6) δ: 21 (CH3), 87.2, 89.4, 110.6, 113.4, 119.5, 123.5, 127.3, 127.6, 129.2, 129.4, 129.6, 139.3, 139.6, 143.2, 144.5, 145.2, 150.2, 150.6, 156.6; MS (*m/z*): 418 (M+, 2), 417 (100), 223 (12), 222 (60), 195 (70), 194 (15), 181 (38), 180 (48), 43 (15); *Anal.* Calcd. for C_26_H_18_N_4_O_2_ (418.45): C, 74.63; H, 4.34; N, 13.39; found: C, 74.84; H, 4.21; N, 13.50.

#### 3,5-Di(furan-2-yl)-4,5-dihydro-*1H*-pyrazole-1-carbothioamide (**5**), Mp: 164–166 °C (lit. mp: 162–163 °C) [35]

##### Ethyl 2-(3,5-di(furan-2-yl)-4,5-dihydro-*1H*-pyrazol-1-yl)-4-methylthiazole-5-carboxylate (**6**)

A mixture of 3,5-di(furan-2-yl)-4,5-dihydro-1*H*-pyrazole-1-carbothioamide (**5**) (2.61 g, 10 mmol) and ethyl 2-chloroacetoacetate (1.38 mL, 10 mmol) was heated under reflux in ethanolic triethylamine for 2 h, then allowed to cool at room temperature. The precipitate formed was filtered off, and recrystallized from ethanol to obtain compound (**6**) as a yellow solid from ethanol, yield (3.15 g, 85%), mp: 140**–**141 **°**C; IR (KBr, cm^−1^): 3120 (=C–H), 2979 (–C–H), 1735 (C=O); ^1^H NMR (CDCl_3_): $\varvec{ }\delta$: 1.29 (t, 3H, CH_2_CH_3_), 2.54 (s, 3H, 4-CH_3_-thiazole), 3.50 (dd, 1H, pyrazoline-H), 3.64 (dd, 1H, pyrazoline-H), 4.21 (q, 2H, CH_2_CH_3_), 5.71 (dd, 1H, pyrazoline-H), 6.29**–**6.30 (d, 1H, furan H-4), 6.39**–**6.40 (t, 1H, furan H-3), 6.52**–**6.55 (t, 1H, furan H-4), 6.81**–**6.82 (d, 1H, furan H-3), 7.32**–**7.33 (d, 1H, furan H-5), 7.55**–**7.57 (d, 1H, furan H-5); ^13^C-NMR (DMSO-*d*6) δ: 14.3, 15.9, 30.2, 41.2, 59.9, 60.9, 96.8, 104.7, 105.0, 105.5, 110.1, 143.6, 144.9, 148.6, 149.7, 49.3, 156.5, 151.9, 164.9. MS (*m/z*): 373 (M+2, 3), 372 (M+1, 23), 371 (M+, 86), 264 (11), 237 (100), 131 (42), 106 (16), 77 (26); Anal. Calcd. for C_18_H_17_N_3_O_4_S (371.41): C, 58.21; H, 4.61; N, 11.31; S, 8.63; found: C, 58.33; H, 4.85; N, 11.16; S, 8.82.

##### 1-(2-(3,5-Di(furan-2-yl)-4,5-dihydro-*1H*-pyrazol-1-yl)-4-methylthiazol-5-yl)-ethanone (**7**)

A mixture of 3,5-di(furan-2-yl)-4,5-dihydro-1*H*-pyrazole-1-carbothioamide (**5**) (2.61 g, 10 mmol), and 3-chloro-2,4-pentanedione (1.13 mL, 10 mmol) was heated under reflux in ethanolic triethylamine for 2 h, then, allowed to cool at room temperature. The precipitate formed was filtered off, and recrystallized from glacial acetic acid to obtain compound (**7**) as a pale yellow solid from glacial acetic acid, yield (2.25 g, 66%), mp: 149**–**151 **°**C; IR (KBr, cm^−1^): 3118 (=C–H aromatic), 2999 (–C–H), 1695 (C=O); ^1^H NMR (CDCl_3_): $\varvec{ }\delta$: 2.41 (s, 3H, 4-CH_3_-thiazole), 2.55 (s, 3H, -COCH_3_), 3.52 (dd, 1H, pyrazoline-H), 3.66 (dd, 1H, pyrazoline-H), 5.72 (dd, 1H, pyrazoline-H), 6.29**–**6.30 (d, 1H, furan H-4), 6.39**–**6.40 (t, 1H, furan H-3), 6.52**–**6.55 (t, 1H, furan H-4), 6.81**–**6.82 (d, 1H, furan H-3), 7.32**–**7.33 (d, 1H, furan H-5), 7.55**–**7.57 (d, 1H, furan H-5); ^13^C-NMR (DMSO-*d*6) δ: 17.1, 28.6, 41.2, 59.9, 104.6, 105.0, 105.6, 109.8, 127.3, 143.7, 177.7, 148.6, 149.2, 155.9, 156.6, 159.9, 189.9. MS (*m/z*): 343 (M+2, 3), 342 (M+1, 22), 341 (M+, 100), 240 (79), 176 (26), 148 (12), 132 (21), 130 (19), 118 (11), 77 (20), 29 (20); *Anal.* Calcd. for C_17_H_15_N_3_O_3_S (341.38): C, 59.81; H, 4.43; N, 12.31; S, 9.39; found: C, 59.78; H, 4.25; N, 12.11; S, 9.48.

##### 2-(3,5-Di(furan-2-yl)-4,5-dihydro-*1H*-pyrazol-1-yl)thiazol-4(*5H*)-one (**8**)

A mixture of 5-di(furan-2-yl)-4,5-dihydro-1H-pyrazole-1-carbothioamide (**5**) (2.61 g, 10 mmol), and ethyl chloroacetate (1.06 mL, 10 mmol) was heated under reflux in ethanolic triethylamine for 2 h, before the reaction mixture was allowed to cool to room temperature. Next, the precipitate formed was filtered off, and recrystallized from dioxane to afford compound (**8**) as a white solid, yield (1.95 g, 65%), mp: 242–245 °C; IR (KBr, cm^−1^): 3150 (=C–H aromatic), 2966 (–C–H), 1694 (C=O); ^1^H NMR (CDCl_3_): $\delta$: 3.67 (dd, 1H, pyrazoline-H), 3.87 (dd, 1H, pyrazoline), 3.89 (s, 2H, thiazolone), 5.88 (dd, 1H, pyrazoline-H), 6.29–6.30 (d, 1H, furan H-4), 6.39–6.40 (t, 1H, furan H-3), 6.52–6.55 (t, 1H, furan H-4), 6.81–6.82 (d, 1H, furan H-3), 7.32–7.33 (d, 1H, furan H-5), 7.55–7.57 (d, 1H, furan H-5); ^13^C-NMR (DMSO-*d*6) δ: 37.6, 41.1, 61.3, 104.7, 105.0, 105.6, 111.3, 143.7, 177.6, 148.6, 149.2, 156.5, 159.8, 182.2. MS (*m/z*): 301 (M+, 3), 182 (20), 143 (11), 139 (21), 129 (17), 128 (10), 117 (27), 115 (39), 96 (16), 75 (19), 43 (100); Anal. Calcd. for C_14_H_11_N_3_O_3_S (301.32): C, 55.80; H, 3.68; N, 13.95; S, 10.64; found: C, 55.70; H, 3.72; N, 14.18; S, 10.53.

##### 2-(3,5-Di(furan-2-yl)-4,5-dihydro-*1H*-pyrazol-1-yl)-4-methylthiazole-5-carbohydrazide (**9**)

A mixture of ethyl 2-(3,5-di(furan-2-yl)-4,5-dihydro-1*H*-pyrazol-1-yl)-4-methylthiazole-5-carboxylate **(6)** (3.71 g, 10 mmol) and 20 mL of hydrazine hydrate was heated under reflux for 12 h, and the reaction mixture allowed to cool at room temperature. Next, the white precipitate was collected, washed with ethanol, and recrystallized from glacial acetic acid to afford compound (**9**); yield (2.32 g, 65%), mp: 212–215 °C; IR (KBr, cm^−1^): 3430 (N–H), 3325, 3273 (NH_2_), 3076 (= C-H), 2930 (–C–H), 1646 (C=O); ^1^H NMR (CDCl_3_): $\delta$: 2.34 (s, 3H, 4-CH_3_-thiazole), 3.41 (dd, 1H, pyrazoline-H), 3.62 (dd, 1H, pyrazoline-H), 5.59 (dd, 1H, pyrazoline-H), 6.29–7.64 (m, 9H, N–H, NH_2_ and furan-H’s); ^13^C-NMR (DMSO-*d*6) δ: 15.4, 41.2, 59.8, 104.8, 105.0, 105.6, 109.2, 121.1, 143.6, 144.7, 148.7, 149.1, 156.3, 156.8, 161.2, 164.8. MS (*m/z*): 358 (M+1, 2), 357 (M+, 11), 182 (16), 181 (100), 166 (36), 165 (11), 151 (38), 135 (24), 120 (17), 107 (29), 89 (16), 79 (32), 73 (38), 71 (11), 63 (11), 45 (91), 44 (12), 43 (38), 31 (14), 29 (16), 28 (23), 27 (16); *Anal.* Calcd. for C_16_H_15_N_5_O_3_S (357.39): C, 53.77; H, 4.23; N, 19.60; S, 8.97; found: C, 53.56; H, 4.34; N, 19.81; S, 9.17.

##### 2-(3,5-Di(furan-2-yl)-4,5-dihydro-*1H*-pyrazol-1-yl)-4-methylthiazole-5-carbonyl azide (**10**)

A sodium nitrite solution (1.38 g, 20 mmol, water (20 mL)) was added portionwise to a suspension solution of 2-(3,5-di(furan-2-yl)-4,5-dihydro-1*H*-pyrazol-1-yl)-4-methylthiazole-5-carbohydrazide (3.57 g, 10 mmol) in hydrochloric acid (20 mL, 6 M) at 0–5 °C with stirring. A brownish yellow precipitate was formed, filtered off, washed with water, and recrystallized from water to afford compound (**10**) as a yellow color with yield (2.69 g, 73%), mp: 162–164 °C; IR (KBr, cm^−1^): 3133 (=C–H), 2927 (–C–H), 2120 (–N_3_), 1635 (C=O); ^1^H NMR (CDCl_3_): $\delta$: 2.50 (s, 3H, 4-CH_3_-thiazole), 3.40 (dd, 1H, pyrazoline-H), 3.83 (dd, 1H, pyrazoline-H), 5.60 (dd, 1H, pyrazoline-H), 6.29–6.30 (d, 1H, furan H-4), 6.39–6.40 (t, 1H, furan H-3), 6.52–6.55 (t, 1H, furan H-4), 6.81–6.82 (d, 1H, furan H-3), 7.32–7.33 (d, 1H, furan H-5), 7.55–7.57 (d, 1H, furan H-5); ^13^C-NMR (DMSO-*d*6) δ: 15.4, 41.1, 59.8, 104.7, 105.1, 1.6.2, 109.3, 111.5, 143.7, 144.6, 148.5, 149.8, 156.4, 158.9, 161.4, 165.0; MS (*m/z*): 369 (M+1, 1), 368 (M+, 5), 327 (12), 326 (60), 311 (19), 309 (19), 284 (23), 283 (14), 256 (17), 255 (100), 43 (14); *Anal.* Calcd. for C_16_H_12_N_6_O_3_S (368.37): C, 52.17; H, 3.28; N, 22.81; S, 8.70; found: C, 52.34; H, 3.15; N, 22.67; S, 8.88.

##### 1-(Aryl)-4-methylthiazol-5-yl)-3-aryl^`^urea (**11a**) and (**11b**)

A mixture of 2-(3,5-di(furan-2-yl)-4,5-dihydro-1*H*-pyrazol-1-yl)-4-methylthiazole-5-carbonyl azide (**10**) (1.94 g, 5 mmol), and the appropriate aniline or 4-methylaniline (5 mmol), was heated under reflux in dioxane (20 mL) for 3 h. The precipitate that formed after cooling at room temperature was collected, and recrystallized from dioxane.

##### 1-(2-(3,5-Di(furan-2-yl)-4,5-dihydro-*1H*-pyrazol-1-yl)-4-methylthiazol-5-yl)-3-phenyl-urea (**11a**)

Pale yellow solid from dioxane, yield (1.62 g, 75%), mp: 191–192 °C; IR (KBr, cm^−1^): 3423 (N–H), 3035 (=C–H aromatic), 2841 (–C–H), 1665 (–CO); ^1^H NMR (CDCl_3_): $\delta$: 2.60 (s, 3H, 4-CH_3_-thiazole), 3.49 (dd, 1H, pyrazoline-H), 3.88 (dd, 1H, pyrazoline-H), 5.89 (dd, 1H, pyrazoline-H), 6.41–7.74 (m, 11H, ArH’s + furan-H’s), 10.72 (s, 2H, 2 N–H); ^13^C-NMR (DMSO-*d*6) δ: 11.5, 41.6, 59.9, 104.5, 105.3, 105.7, 106.4, 119.2, 121.7, 123.6, 125.5, 129.2, 138.3, 143.6, 144.3, 148.6, 149.3, 152.6, 156.6, 166.1; MS (*m/z*): 433 (M+, 1), 279 (12), 278 (75), 277 (44), 262 (20), 247 (10), 283 (17), 281 (24), 122 (10), 79 (14), 91 (14), 79 (14), 78 (17), 77 (27), 75 (19), 57 (23), 28 (100); *Anal.* Calcd. for C_22_H_19_N_5_O_3_S (433.48): C, 60.96; H, 4.42; N, 16.16; S, 7.40; found: C, 61.14; H, 4.28; N, 16.00; S, 7.45.

##### 1-(2-(3,5-Di(furan-2-yl)-4,5-dihydro-*1H*-pyrazol-1-yl)-4-methylthiazol-5-yl)-3-(p-tolyl)-urea (**11b**)

White solid from dioxane, yield (1.56 g, 70%), mp: 238–241 °C; IR (KBr, cm^−1^): 3432 (N–H), 3025 (=C–H aromatic), 2914 (–C–H), 1624 (–C = O); ^1^H NMR (CDCl_3_): $\delta$: 2.35 (s, 3H, 4-CH_3_C_6_H_4_), 2.50 (s, 3H, 4-CH_3_-thiazole), 3.51 (dd, 1H, pyrazoline-H), 3.88 (dd, 1H, pyrazoline-H), 5.78 (dd, 1H, pyrazoline-H), 6.43–8.29 (m, 10H, ArH’s + furan-H’s), 10.73 (s, 2H, 2 N–H); ^13^C-NMR (DMSO-*d*6) δ: 11.8, 20.6, 41.1, 58.8, 104.6, 105.0, 105.9, 109.1, 121.6, 122.5, 125.4, 129.6, 131.9, 137.8, 143.7, 144.7, 148.5, 149.1, 151.8, 156.6, 165.8. MS (*m/z*): 447 (M+, 1), 411 (10), 380 (13), 232 (29), 191 (22), 190 (17), 189 (100), 162 (16), 134 (22), 43 (10); *Anal.* Calcd. for C_23_H_21_N_5_O_3_S (447.51): C, 61.73; H, 4.73; N, 15.65; S, 7.17; found: C, 61.76; H, 4.84; N, 15.72; S, 7.32.

##### 3-(2-(3,5-Di(furan-2-yl)-4,5-dihydro-*1H*-pyrazol-1-yl)-4-methylthiazol-5-yl)quinazoline-2,4(*1H,3H*)-dione (**12**)

*Method A* A mixture of 2-(3,5-di(furan-2-yl)-4,5-dihydro-1*H*-pyrazol-1-yl)-4-methylthiazole-5-carbonyl azide (**10**) (1.94 g, 5 mmol) and anthranilic acid (0.68 g, 5 mmol) was heated under reflux in dioxane (20 mL) for 4 h. The solid that formed after the reaction mixture was cooled and recrystallized from glacial acetic acid to realize compound (**12**).

*Method B* A mixture of 2-(3,5-di(furan-2-yl)-4,5-dihydro-1H-pyrazol-1-yl)-4-methylthiazole-5-carbonyl azide (10) (1.94 g, 5 mmol) and methyl anthranilate (0.75 g, 5 mmol) was heated under reflux in dioxane (20 mL) for 4 h. The solid that formed after the reaction mixture was cooled and recrystallized from glacial acetic acid produced a product identical in all respects (mp, mixed mp, and spectra) with compound (**12**). White solid from glacial acetic acid, yield (1.51 g, 66%), mp: 161–162 °C; IR (KBr, cm^−1^): 3415 (N–H), 3154 (=C–H aromatic), 3046 (=C–H), 2950 (–C–H), 1643 (CO); ^1^H NMR (CDCl_3_): $\varvec{ }\delta$: 2.34 (s, 3H, 4-CH_3_C_6_H_4_), 3.43–3.52 (dd, 1H, *J* = 12 Hz, pyrazoline CH), 3.81–3.90 (dd, 1H, *J* = 12 Hz, pyrazoline CH), 5.66–5.71 (dd, 1H, J = 12 Hz, pyrazoline CH), 6.12–8.17 (m, 10H, ArH’s) and 10.6 (s, br., 1H, NH); ^13^C-NMR (DMSO-*d*6) δ: 12.1, 41.3, 59.8, 104.7, 105.0, 105.8, 109.1, 114.9, 117.2, 123.2, 114.9, 126.9, 135.4, 136.2, 139.8, 143.6, 144.6, 147.1, 148.6, 149.2, 159.5, 157.4, 163.8. MS (*m/z*): 459 (M+, 2), 300 (8), 256 (9), 256 (11), 225 (12), 161 (12), 147 (32), 136 (20), 134 (13), 123 (15), 122 (100), 121 (74), 119 (11), 107 (14), 91 (20), 77 (10), 56 (10), 55 (17), 43 (12), 41 (10), 28 (17); *Anal.* Calcd. for C_23_H_17_N_5_O_4_S (459.48): C, 60.12; H, 3.73; N, 15.24; S, 6.98; found: C, 60.22; H, 3.65; N, 15.10; S, 7.11.

##### Naphthalen-2-yl(2-(3,5-di(furan-2-yl)-4,5-dihydro-*1H*-pyrazol-1-yl)-4-methylthiazol-5-yl)carbamate (**13**)

A mixture of 2-(3,5-di(furan-2-yl)-4,5-dihydro-1*H*-pyrazol-1-yl)-4-methylthiazole-5-carbonyl azide **(10)** (1.94 g, 5 mmol) and 2-naphthol (0.72 g, 5 mmol), was heated under reflux in dry benzene (20 mL). The reaction mixture was allowed to cool at room temperature, then the precipitate that formed was collected and recrystallized from glacial acetic acid to afford compound (**13**) as a white solid from glacial acetic acid, yield (1.93 g, 80%), mp: 225–227 °C; IR (KBr, cm^−1^): 3432 (N-H), 3115 (=C–H aromatic), 2811 (–C–H), 1603 (C=O); ^1^H NMR (CDCl_3_): $\varvec{ }\delta$: 2.49 (s, 3H, 4-CH_3_-thiazole), 3.34 (dd, 1H, pyrazoline-H), 3.73 (dd, 1H, pyrazoline-H), 5.56 (dd, 1H, pyrazoline-H), 6.39-8.09 (m, 13H, ArH’s + furan-H’s), 10.2 (s, 1H, N-H); ^13^C-NMR (DMSO-*d*6) δ: 11.5, 41.6, 59.9, 105.3, 105.7, 109.2, 111.4, 113.3, 122.8, 128.7, 125.2, 125.6, 126.4, 128.9, 129.8, 134.7, 136.7, 143.5. 144.8, 148.4, 148.8, 156.8, 166.0; MS (*m/z*): 485 (M+1, 1), 484 (M+, 3), 422 (10), 403 (16), 402 (22), 360 (11), 319 (31), 318 (100), 275 (12), 274 (60), 273 (12), 225 (18), 121 (13), 85 (40), 57 (53), 43 (11), 41 (11); *Anal.* Calcd. for C_26_H_20_N_4_O_4_S (484.53): C, 64.45; H, 4.16; N, 11.56; S, 6.62; found: C, 64.53; H, 4.23; N, 11.68; S, 6.77.

#### 5-Amino-2-aryl-7-aryl`-*7H*-pyrano[2,3-*d*]thiazole-6-carbonitrile (**15a**, **b**)

*Method A* A mixture of 2-(3,5-di(furan-2-yl)-4,5-dihydro-1*H*-pyrazol-1-yl)thiazol-4(5*H*)-one (1.5 g, 5 mmol), and the appropriate arylidene malononitrile **(14a**) or (**14b**) was heated under reflux in ethanol (20 mL) containing a catalytic amount of piperidine for 2 h. The solid so formed after the reaction mixture was cooled to room temperature was collected and recrystallized from dioxane to yield compounds (**15a**, and **15b**), respectively.

*Method B* A mixture of 2-(3,5-di(furan-2-yl)-4,5-dihydro-1*H*-pyrazol-1-yl)thiazol-4(5*H*)-one (1.5 g, 5 mmol), the appropriate amount of benzaldehyde or 4-methoxybenzaldehyde (5 mmol), malononitrile (0.33 g, 5 mmol) and piperidine (0.42 g, 5 mmol) in 20 mL ethanol was heated under reflux for 2 h. The solid that formed after the reaction mixture was cooled to room temperature was collected and recrystallized from dioxane to yield compounds identical in all aspects (mp, mixed mp and spectra) with the product obtained in method A.

##### 5-Amino-2-(3,5-di(furan-2-yl)-4,5-dihydro-*1H*-pyrazol-1-yl)-7-phenyl-*7H*-pyrano[2,3-*d*]thiazole-6-carbonitrile (**15a**)

Pale yellow solid from dioxane, yield (1.48 g, 65%), mp: 295–296 °C; IR (KBr, cm^−1^): 3320, 3270 (NH_2_), 3056 (–C–H), 2988 (–C–H), 2278 (–CN); ^1^H NMR (CDCl_3_): $\delta$: 3.56 (dd, 1H, pyrazoline-H), 4.02 (dd, 1H, pyrazoline-H), 5.11 (dd, 1H, pyrazoline-H), 5.50 (s, 1H, pyran H-4), 6.22 (s, 2H, NH_2_), 6.80–7.63 (m, 11H, ArH’s + furan-H’s); ^13^C-NMR (DMSO-*d*6) δ: 34.1, 38.2, 59.9, 92.6, 104.8, 105.0, 109.1,125.7, 128.8, 129.1, 142.8, 143.3, 143.6, 144.7, 149.5, 149.3, 154.2, 155.4, 156.1, 156.6. MS (*m/z*): 456 (M+1, 3), 455 (M+, 12), 382 (17), 319 (22), 318 (100), 290 (33), 151 (19), 128 (14); Anal. Calcd. for C_24_H_17_N_5_O_3_S (455.49): C, 63.29; H, 3.76; N, 15.38; S, 7.04; found: C, 63.38; H, 3.67; N, 15.16; S, 7.20.

##### 5-Amino-2-(3,5-di(furan-2-yl)-4,5-dihydro-*1H*-pyrazol-1-yl)-7-(4-methoxyphenyl)-*7H*-pyrano[2,3-*d*]thiazole-6-carbonitrile (**15b**)

Pale yellow solid from dioxane, yield (1.62 g, 67%), mp: 304–307 °C; IR (KBr, cm^−1^): 3320, 3273 (NH_2_), 3070 (–C=H), 2986 (–C–H), 2228 (–CN); ^1^H NMR (CDCl_3_): $\delta$: 3.52 (dd, 1H, pyrazoline-H), 3.84 (s, 3H, -OCH_3_), 3.96 (dd, 1H, pyrazoline-H), 5.12 (dd, 1H, pyrazoline-H), 5.55 (s, 1H, pyran-H), 6.22 (s, 2H, NH_2_), 6.45–7.62 (m, 10H, ArH’s + furan-H’s); ^13^C-NMR (DMSO-*d*6) δ: 34.3, 36.5, 41.3, 56.2, 59.8, 92.7, 104.5, 105.7, 106.1, 116.4, 131.5, 134.8, 142.8, 143.6, 144.8, 148.4, 148.9, 154.2, 155.2, 155.7, 156.3, 165.5; MS (*m/z*): 485 (M + , 5), 478 (24), 477 (87), 446 (25), 445 (100), 399 (24), 396 (20), 373 (22), 372 (25), 327 (41), 326 (24), 251 (10); *Anal.* Calcd. for. for C_25_H_19_N_5_O_4_S (485.51): C, 61.85; H, 3.94; N, 14.42; S, 6.60; found: C, 61.73; H, 4.13; N, 14.35; S, 6.76.

### Evaluation of the antitumor activity using viability Assay

Crystal violet stain (1%), composed of 0.5% (w/v) crystal violet and 50% methanol, was made up to volume with ddH_2_O and filtered through a Whitman No. 1 filter paper.

### Cytotoxicity evaluation using viability assay

Human hepatocellular breast (MCF-7) and colon (HCT-116) carcinoma cells were obtained from the VACSERA Tissue Culture Unit. The cells were propagated in Dulbecco’s modified Eagle’s medium (DMEM), and supplemented with 10% heat-inactivated fetal bovine serum, 1% l-glutamine, HEPES buffer and 50 μmol mL^−1^ gentamycin. All cells were maintained at 37 °C in a humidified atmosphere with 5% CO_2_ and were sub-cultured twice a week.

### Evaluation of cytotoxicity activity

Cytotoxicity of all compounds was tested in MCF-7 and HCT-116 cells. All experiments and data concerning the cytotoxicity evaluation were performed at the Regional Center for Mycology and Biotechnology RCMB, Al-Azhar University, Cairo, Egypt. For the cytotoxicity assay, cells were seeded in a 96-well plate at a cell concentration of 1 × 10^4^ cells per well in 100 μL of growth medium. Fresh medium containing different concentrations of the test sample was added after 24 h of seeding. Serial two-fold dilutions of the tested compounds were added to confluent cell monolayers dispensed into 96-well, flat-bottomed microtiter plates (Falcon, NJ, USA) using a multichannel pipette. The microtiter plates were incubated at 37 °C in a humidified incubator with 5% CO_2_ for a period of 48 h. Three wells were used for each concentration of the test sample. Control cells were incubated without the test sample and with or without DMSO. The little percentage of DMSO present in the wells (maximal 0.1%) was found not to affect the experiment. After incubation of the cells for at 37 °C, various concentrations of the sample were added, and the incubation continued for 24 h before viable cell yield was determined using a colorimetric method. In brief, after the end of the incubation period, media were aspirated and the crystal violet solution (1%) was added to each well for at least 30 min. The stain was removed and the plates were rinsed using tap water until all excess stain was removed. Glacial acetic acid (30%) was then added to all wells and mixed thoroughly, before the absorbance of the plates was measured (after being gently shaken) on a Microplate reader (TECAN, Inc.), using a test wavelength of 490 nm. All results were corrected for background absorbance detected in wells without added stain. Treated samples were compared with the cell control in the absence of the tested compounds. All experiments were carried out in triplicate. The cell cytotoxic effect of each tested compound was calculated. Optical density was measured with a microplate reader (SunRise, TECAN, Inc., USA) to determine the number of viable cells, and the percentage of viability was calculated as the percentage of cell viability = [1 − (ODt/ODc)] × 100% where ODt is the mean optical density of wells treated with the tested sample and ODc is the mean optical density of untreated cells. The relationship between the surviving cells and drug concentration was plotted to obtain the survival curve of each tumor cell line after treatment with the specified compound. The 50% inhibitory concentration (IC_50_), the concentration required to cause toxic effects in 50% of intact cells, was estimated from graphic plots of the dose response curve for each concentration using Graphpad Prism software (San Diego, CA. USA).

## Antimicrobial activity assay

Chemical compounds under investigation were individually tested against a panel of Gram-positive and Gram-negative bacterial pathogens, and fungi. Antimicrobial tests were conducted using the agar well-diffusion method [36–38]. After the media had cooled and solidified, wells (6 mm in diameter) were made in the solidified agar, before microbial inoculum was uniformly spread using a sterile cotton swab on a sterile Petri dish containing nutrient agar (NA) medium, or Sabouraud dextrose agar (SDA) media for bacteria and fungi, respectively. An amount of 100 µL of the tested compound solution was prepared by dissolving 1 mg of the compound in 1 mL of dimethylsulfoxide (DMSO). The inoculated plates were then incubated for 24 h at 37 °C for bacteria and yeast, and 48 h at 28 °C for fungi. Negative controls were prepared using DMSO employed for dissolving the tested compound. Amphotericin B (1 mg/mL), Ampicillin (1 mg/mL), and Gentamicin (1 mg/mL) were used as standards for bacteria and fungi, respectively. After incubation, antimicrobial activity was evaluated by measuring the zone of inhibition against the tested microorganisms. Antimicrobial activity was expressed as inhibition diameter zones in millimeters (mm).

## Conclusions

In summary, new and efficient synthetic routes of some prepared pyridines, pyrazoline, thiazoles, and pyrano[2,3-*d*]thiazole were achieved. The structure of the newly prepared compounds was established based on elemental analysis, spectral data, and alternative methods wherever possible. The synthesized compounds (**3a**, **4a**, **4d**–**4f**, **5**, **7**–**9**, **11a**, and **11b**) were investigated against two carcinoma cell lines: breast MCF-7 and colon HCT-116 human cancer cell lines. Our results showed that compounds **4e**, **4f**, **11a**, and **11b** had the lowest IC_50_ values against HCT-116 cancer cells. In addition, the selected newly prepared compounds were evaluated for their antimicrobial activity against Gram-positive and Gram-negative bacteria as well as some fungal-plants. The results proved that some prepared compounds showed an adequate inhibitory efficiency of growth of Gram-positive and Gram-negative bacteria.

## Abbreviations

HCT-116: human cancer cell lines
MCF-7: estrogen responsive proliferative breast cancer model
DMEM: Dulbecco’s modified Eagle’s medium
HIV: human immunodeficiency virus
IC_50_: the concentration of an inhibitor that is required for 50-percent inhibition of an enzyme in vitro
mp: melting point
Mw: molecular weight
